# Chitinase producing bacteria with direct algicidal activity on marine diatoms

**DOI:** 10.1038/srep21984

**Published:** 2016-02-23

**Authors:** Yi Li, Xueqian Lei, Hong Zhu, Huajun Zhang, Chengwei Guan, Zhangran Chen, Wei Zheng, Lijun Fu, Tianling Zheng

**Affiliations:** 1 State Key Laboratory of Marine Environmental Science and Key Laboratory of MOE for Coast and Wetland Ecosystems, School of Life Sciences, Xiamen University, Xiamen 361005, China; 2 College of Life Sciences, Henan Normal University, Xinxiang, 453007, China; 3 Department of Environment and Life Science, Putian University, Putian 351100, China; 4 Tobacco Science Institute of Jiangxi Province, Nanchang 330000, China

## Abstract

Chitinase producing bacteria can involve extensively in nutrient cycling and energy flow in the aquatic environment through degradation and utilization of chitin. It is well known that diatoms cells are encased by box-like frustules composed of chitin. Thus the chitin containing of diatoms shall be a natural target of chitinase producing bacteria, however, the interaction between these two organismic groups has not been studied thus far. Therefore, in this study, the algicidal mechanism of one chitinase producing bacterium (strain LY03) on *Thalassiosira pseudonana* was investigated. The algicidal range and algicidal mode of strain LY03 were first studied, and then bacterial viability, chemotactic ability and direct interaction characteristic between bacteria and diatom were also confirmed. Finally, the characteristic of the intracellular algicidal substance was identified and the algicidal mechanism was determined whereby algicidal bacterial cells showed chemotaxis to algal cells, fastened themselves on algal cells with their flagella, and then produced chitinase to degrade algal cell walls, and eventually caused algal lysis and death. It is the first time to investigate the interaction between chitinase producing bacteria and diatoms, and this novel special interaction mode was confirmed in this study, which will be helpful in protection and utilization of diatoms resources.

Chitin is the most abundant natural polysaccharide which is combined by N-acetylglucosamine through a *β*, 1–4-linked polymer^1^, and is extracted primarily from shellfish sources. In the marine environment, quantities of chitin are produced and accumulated annually, which are rich, important nutrient and energy source^2^. The degradation and utilization of chitin by many chitinase producing bacteria is a key nutrient cycling and energy flow in the marine environment^3^. Many bacteria can use chitin as the sole source of C and N^4^, and the chitin is degraded into oligosaccharides or monosaccharides by chitinase, which then flow into the microbial loop and are absorbed by other organisms. Marine phytoplankton account for the major primary production and have a strong influence on global nutrient cycling^5^, and some members of the phytoplankton are protected by cell walls containing chitin, especially the marine diatoms.

Marine diatoms supply ~40% of marine primary productivity, serve as the base of the marine food web^6^, and are encased in distinctive, porous silica shells, called frustules^7^. The frustules of diatoms are one of the most important components of the diatom cell, and all diatoms except *Phaeodactylum tricornutum* contain chitin in their frustules^8^. Heterotrophic bacteria with high abundance and diversity are ubiquitous scavengers that utilize organic carbon produced by diatoms and other autotrophs, and always pose a serious threat to diatoms^9^. Frustules can protect diatoms against the algicidal effect of many heterotrophic bacteria. However, whether or not the silica frustules containing chitin are degraded by chitinase producing bacteria is unclear. To better determine the functional mechanism of chitinase producing bacteria and to protect diatom resources, the interaction between a chitinase producing bacteria and a diatom (*Thalassiosira pseudonana*) were investigated by means of performed to carry out in-depth research.

Understanding interactions between chitinase producing bacteria and diatoms is of primary importance to deciphering oceanic nutrient cycles and energy fluxes. In this study, the chitinase producing bacterium, *Chitinimonas prasina* LY03, which can produce chitinase to degrade diatom frustules was used to investigate the interaction between bacterium and diatom. The algicidal range, algicidal activity and algicidal mode were first determined, and then bacterial viability, chemotactic ability and the direct contact characteristic between bacteria and diatoms were used to confirm direct algicidal activity. Next, the characteristics of the intracellular algicidal material were studied to determine the chitinase activity. Finally, we set out to confirm the direct algicidal activity of chitinase producing bacteria on diatoms.

## Materials and Methods

### Growth and maintenance of *C*. *prasina* LY03 and *C. koreensis* KACC 11467

*C*. *prasina* LY03, which was deposited in the Marine Culture Collection of China with accession number MCCC 1F01209^10^ and *C. koreensis* KACC 11467, which was purchased from the Korean Agricultural Culture Collection, were grown in nutrient agar (NA; Difco) (distilled water 1 L, peptone 5.0 g, meat extract 3.0 g, agar 15.0 g, pH 7.2). Cell cultures were grown at 37 °C for 24 h.

### Algal cultures

The experimental diatom included *Thalassiosira pseudonana*, *Thalassiosira weissflogii*, *Chaetoceros muelleri*, *Skeletonema costatum*, *Phaeodactylum tricornutum*. In addition we have studied: chlorophytes *Dunaliella salina* and *Chlorella autotrophica*; chrysophytes *Phaeocystis globosa*, *Nannochloropsis* sp.; and the xanthophyta *Heterosigma akashiwo*. The above algal strains have been provided by the College of Ocean and Earth Sciences, Xiamen University, Xiamen, China, the dinoflagellate *Scrippsiella trochoidea* and *Alexandrium tamarense* were supplied by the Algal Culture Collection, Institute of Hydrobiology, Jinan University (Guangzhou, China). The algal cultures were cultivated in f/2 medium prepared with natural sea water, which had been passed through a 0.45 μm filter at 20 ± 1 °C under a 12 h: 12 h light-dark cycle with a light intensity of 50 μmol photons m^−2^s^−1^ 11.

### Determination of algicidal activity and algicidal mode

To determine the algicidal activity of *C*. *prasina* LY03 and *C. koreensis* KACC 11467 on experimental algae, the fluorescence intensity of the algal cultures were monitored. Strain LY03 and KACC11467 were inoculated into 20 mL NA medium and grown to the stationary phase at 37 °C in a shaker at 120 rpm for 24 h. To study the algicidal activity, different concentrations of bacterial cultures (0.3, 0.5, 1.0, 2.0, 3.0, 4.0 and 5.0%; v/v) were added into a *T. pseudonana* culture, which was used to determine the algicidal activity and experimental concentration of the bacterial culture. To study the algicidal mode, different treatments were implemented as follows: (1) adding concentrations of 2.0% bacterial culture into algal cultures; (2) adding concentrations of 2.0% of 0.22-μm Millipore membrane bacterial culture filtrate into algal cultures; (3) adding concentrations of 2.0% washed bacterial cells, which were resuspended in sterile f/2 after centrifugation at 10,000 rpm for 5 min and washing twice with sterile f/2 medium into algal cultures, to determine if algicidal activity was from the bacterial cells. The control group was normal growth algae with sterile NA or sterile f/2 medium added to avoid the medium influence. The fluorescence intensity in different treatment groups and control groups was measured under a microplate reader with 440 nm of excitation light and 680 nm of emission light after exposure.

The algicidal rate was calculated using the equation 1:


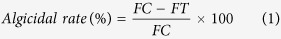


where *FT* is the fluorescence intensity of the treated algal culture and *FC* the fluorescence intensity of the control algal culture^12^.

### Identification of bacterial viability

*C*. *prasina* LY03 was cultured in NA medium at 37 °C for 24 h, and then washed bacterial cells after centrifugation at 10,000 rpm for 5 min as well as washing twice with sterile f/2 medium were added to sterile f/2 medium for 24, 48 and 72 h. Samples in different treatment groups were incubated at 25 °C for 30 min and stained with 10 μg/mL propidium iodide (PI) in dark for 15 min^13^ and then resuspended in f/2 medium. Flow cytometry (FCM) analysis was performed using a BD LSRFortessa cell analyzer (BD, USA), equipped with an arc lamp as the light source. For each sample about 10,000 cells were analyzed.

### Determination of chemotactic ability

f/2 agar medium was prepared by adding 0.18% agar into f/2 medium followed by sterilization. 10 mL of *C*. *prasina* LY03 and KACC11467 cultures were centrifuged at 10,000 rpm for 5 min and washed twice with sterile f/2 medium, before the bacterial cells were resuspended in 500 μL sterile f/2 medium. The bacterial suspension was mixed in the f/2 agar medium after the medium was cooled to room temperature, and then agar plates with bacterial cells were prepared using the pour plate method. 50 mL of *T*. *pseudonana* and *P*. *tricornutum* algal cultures were collected after centrifugation at 3500 rpm for 3 min and washed twice with sterile f/2 medium. After the pellets were resuspended in 50 μL sterile f/2 medium, a small amount of these freshly prepared algal cells was dropped on to the center of the f/2 agar medium containing bacterial cells. A chemotactic ring was observed around the algal lipid droplet after this agar medium was cultured at 37 °C for 24 h.

### Analysis of the interaction between bacterial cells and algae

20 mL of *C. prasina* LY03 and KACC11467 cultures were centrifuged at 10,000 rpm for 5 min and, after washing twice with sterile f/2 medium, bacterial cells of strain LY03 and KACC11467 were added into the axenic exponentially growing algal cultures (*T*. *pseudonana* and *P*. *tricornutum*) at a ratio of 2.0% (v/v). A 10 mL culture was collected (3000 rpm, 5 min, 20 °C), then fixed in 0.1 M sodium phosphate buffer solution (PBS, 8g NaCl, 0.2g KCl, 1.44g Na_2_HPO_4_, 0.24g KH_2_PO_4_, 1 L distilled water, 50 mM, pH7.4) containing 2.5% glutaraldehyde (v/v) for 2 h and then gently rinsed twice with PBS buffer followed by post fixation in 1% OsO_4_ in the same buffer for 2 h. The samples were then gently rinsed twice with PBS buffer followed by dehydration in a graded ethanol series (30, 50, 70, 90, 95 and 100%) and finally stored in pure tertiary butanol at 4 °C overnight. The samples were subsequently critical-point-dried and mounted on stubs. The preparation was sputter coated with gold-palladium at 60:40 and 25:30 nm. The algicidal procedure was visualized and imaged using scanning electron microscopy (SEM, model JSM-6390, JEOL).

### Cell structure changes of *T*. *pseudonana*

Algal cells were treated with bacterial cells for 6, 24, 48 and 72 h, and were then prepared for transmission electron microscopy (TEM). A 20 mL culture was collected (3000 rpm, 5 min, 20 °C), then fixed with 2.5% glutaraldehyde (v/v) for 2–4 h, then washed with 0.1 M PBS twice. Samples were embedded in araldite resin. Sections (60–80 nm), obtained with an ultramicrotome, were stained in 3% acetic acid uranium-citric acid and viewed using TEM (model JEM-2100HC; JEOL)

### Location of algicidal substance in algicidal strain LY03

To evaluate the algicidal activity of each cellular fraction of strain LY03 against *T*. *pseudonana*, each fraction was prepared using the method of Jung^14^, with slight modifications. The bacterial cells of strain LY03 grown in 1 L of NA media were centrifuged at 10,000 rpm for 5 min, the remaining bacterial cells were washed twice with sterile f/2 medium, and then re-suspended with 20 mL of a freshly prepared isotonic buffer solution (TMS) (Tris–HCl 50 mmol L^−1^ pH = 8.0, MgCl_2_ 16 mmol L^−1^, sucrose 33% w/v, lysozyme 100–200 μg L^−1^, phenylmethylsulfonyl fluoride 0.1 mmol L^−1^). The cell suspension was incubated at 37 °C for 30–60 min and centrifuged at 21,000 g at 4 °C for 15 min. Then, the supernatant was carefully collected to obtain periplasmic proteins. The remaining pellet was re-suspended in lysis buffer (Tris–HCl 50 mmol L^−1^ pH = 8.0, MgSO_4_ 5 mmol L^−1^) followed by repeated sonication at 50 A and 4 °C for 5 min. The solution was centrifuged at 21,000 g at 4 °C for 1 h to obtain the cytoplasm in the supernatant. The last remaining pellet was immediately re-suspended in TMS and acquired the cytoplasmic membrane. The algicidal activity of each fraction of strain LY03 was determined following the above method.

### Characteristic identification of intracellular algicidal substances

Intracellular cellular culture of algicidal strain LY03 was obtained using the above-mentioned method, and then different treatments were performed on intracellular substances, in order to determine the characteristic of the intracellular algicidal substances. The algicidal activity of intracellular algicidal substances were studied after the intracellular substances were treated with protease, different temperatures (−80, 25, 55, 65, 80, 90 and 99 °C) and different pH values (1, 2, 3, 7.8, 11, 12 and 13). Intracellular substances were obtained from bacterial cells of strain LY03. The bacterial cells were lysed by sonication at 50 A and 4 °C for 5 min, centrifuged at 10,000 rpm for 5 min, and the intracellular substances were in the intracellular supernatant. The intracellular supernatant was treated under different temperatures for 1–2 h, and added into an algal culture after cooling to room temperature. The pH of the intracellular supernatant was adjusted to different pH values, kept for several hours, and then adjusted back to the initial pH to investigate the algicidal activity.

### Statistics

All data were presented as means ± standard error of the mean and were evaluated using one-way analysis of variance followed by the least significant difference test, with *p* < 0.01 and *p* < 0.05 (Origin 8.5 for Windows).

## Results

### Algicidal activity, algicidal range and algicidal mode of strain LY03

**Figure 1:**
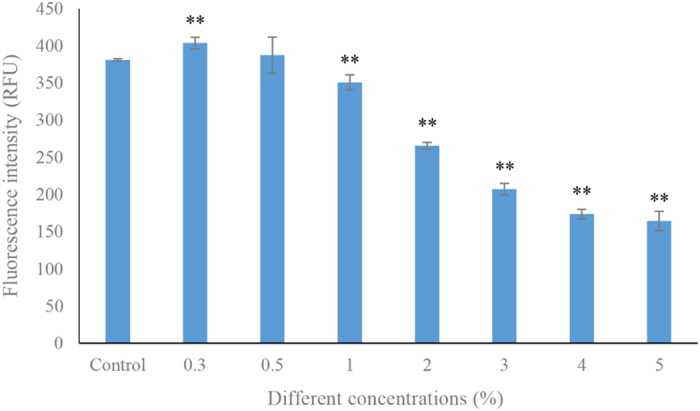
The algicidal rates in different concentrations of bacterial cultures. All error bars indicate the SE of the three biological replicates. ** represents a statistically significant difference of *p* < 0.01 when compared to the control.

Different concentrations of strain LY03 (0.3, 0.5, 1.0, 2.0, 3.0, 4.0 and 5.0%) were added into algal cultures and the algicidal activity was measured in Fig. 1. There was no obvious algicidal activity in concentrations of 0.3 and 0.5% compared to the control. When the amount exceeded concentration of 1.0%, the fluorescence intensity in the treatment groups has significantly (*p* < 0.01) decreased compared to the control, and the algicidal rates in concentrations of 1.0, 2.0, 3.0, 4.0 and 5.0% were 7.9, 30.3, 45.6, 54.4 and 56.8%, respectively. Therefore, we chose a concentration of 2.0% as the experimental concentration for strain LY03.

**Figure 2:**
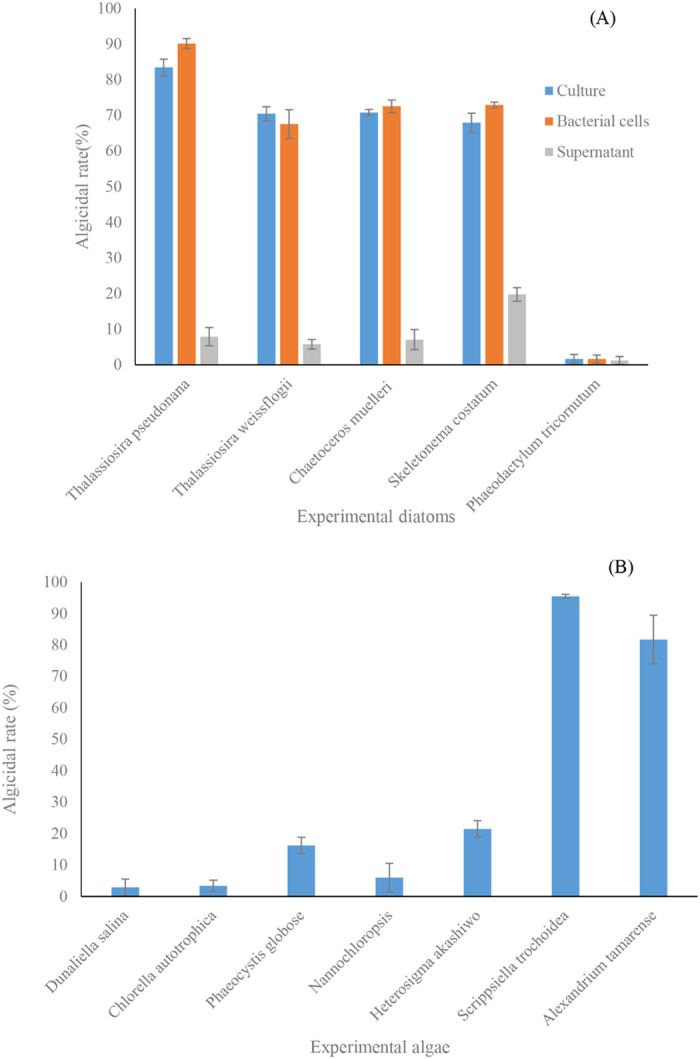
Algicidal range and algicidal mode of strain LY03. All error bars indicate the SE of the three biological replicates.

The algicidal range and algicidal mode of strain LY03 are shown in Fig. 2. To investigate the algicidal effect of LY03 on diatoms, we chose five diatoms: *T. pseudonana*, *T. weissflogii*, *C. muelleri* and *S. costatum*, which contain chitin in their siliceous shells, and *P. tricornutum*, which does not contain a siliceous shell with chitin, as the target algae. Figure 2A showed that all the experimental diatoms except *P. tricornutum* were seriously damaged under the algicidal effect, and the algicidal rate reached 70% after 24 h treatment. We also studied the algicidal activity of strain LY03 on other algae (Fig. 2B), and strain LY03 did not show any algicidal effect on algae other than the dinoflagellates.

To determine the algicidal mode, we separated bacterial cells and the supernatant from bacterial culture, and then investigated the algicidal effect of different fractions (Fig. 2A). The results showed that the bacterial cells of strain LY03 showed high algicidal activity, as well as an obvious algicidal rate of bacterial cells, whereas the supernatant of strain LY03 did not exhibit algicidal effect on any of the experimental algae.

In follow-up experiments, we chose bacterial cells to study the interaction property between bacteria and algae. *T. pseudonana* with chitin and *P. tricornutum* without chitin in algal cells were chosen as experimental algae, because of their advantages as model diatom species and their clear genomic information.

### Bacterial viability in algal cultures

**Figure 3:**
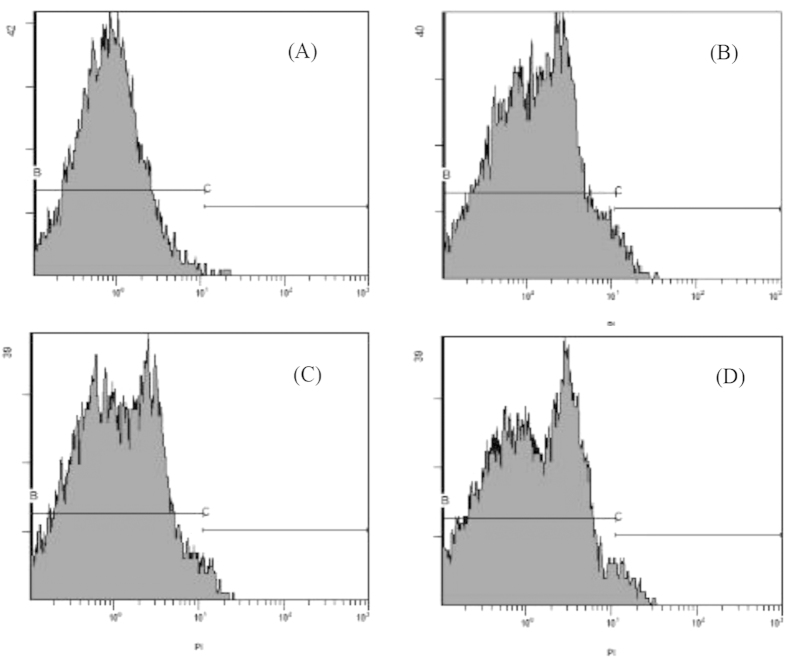
Bacterial viability of strain LY03 after culture in algal cultures for 0 h (**A**), 24 h (**B**), 48 h (**C**) and 72 h (**D**).

To study the viability of strain LY03 after bacterial cells were added into the algal culture, bacterial cells with PI staining were used for study with FCM (Fig. 3). As shown in Fig. 3A, only 0.12% of the bacterial cells in the control were stained with PI, and most of the bacteria survived. After strain LY03 was added to algal cells cultured for 24, 48 and 72 h, the ratios of bacterial cells stained with PI were only 4.39, 3.17 and 4.31%, respectively.

### Chemotactic ability of strain LY03

**Figure 4:**
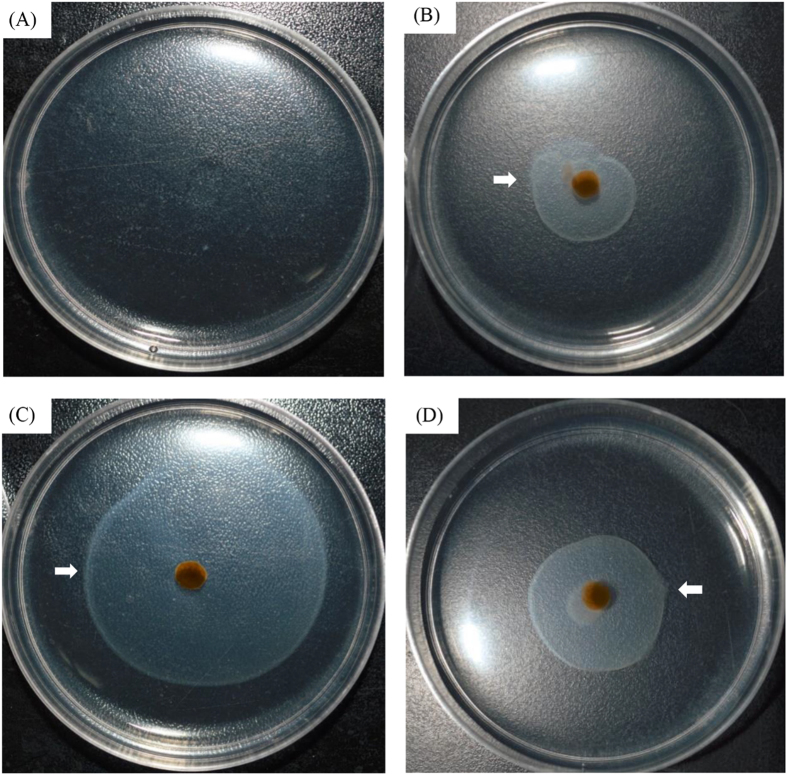
Chemotactic ability after culture at 37 °C for 48 h of strain LY03 with f/2 (**A**), *Thalassiosira pseudonana* (**B**), and *Phaeodactylum tricornutum* (**C**) as chemotactic substances; and of strain KACC 11467 with *Thalassiosira pseudonana* (**D**) as the chemotactic substance.

*C. koreensis* KACC 11467 belongs to the same genus with strain LY03, and can also secrete chitinase. However, strain KACC 11467 showed no algicidal effect on diatoms (Fig. S1), no matter whether bacterial culture, bacterial cells or supernatant. Therefore, strain KACC 11467 was chosen as another chitinase producing bacterium to clarify the functional mechanism between strain LY03 and diatoms. To understand the chemotactic ability of strain LY03 and KACC 11467, chemotaxis plate test were performed in Fig. 4. The results showed that there was an obvious chemotactic ring in the f/2 plate after adding bacterial cells of strain LY03, no matter what the chemotactic substance was. Strain LY03 showed high chemotactic ability to *T. pseudonana* and *P. tricornutum* cells (Fig. 4B,C). We also studied the chemotactic ability of strain KACC 11467 on *T. pseudonana* cells, as shown in Fig. 4D, and there was an obvious chemotactic ring in the plate within strain KACC 11467 cells, which implied that strain KACC 11467 also showed chemotactic ability on *T. pseudonana* cells.

### Interaction between chitinase producing bacteria and diatoms

**Figure 5:**
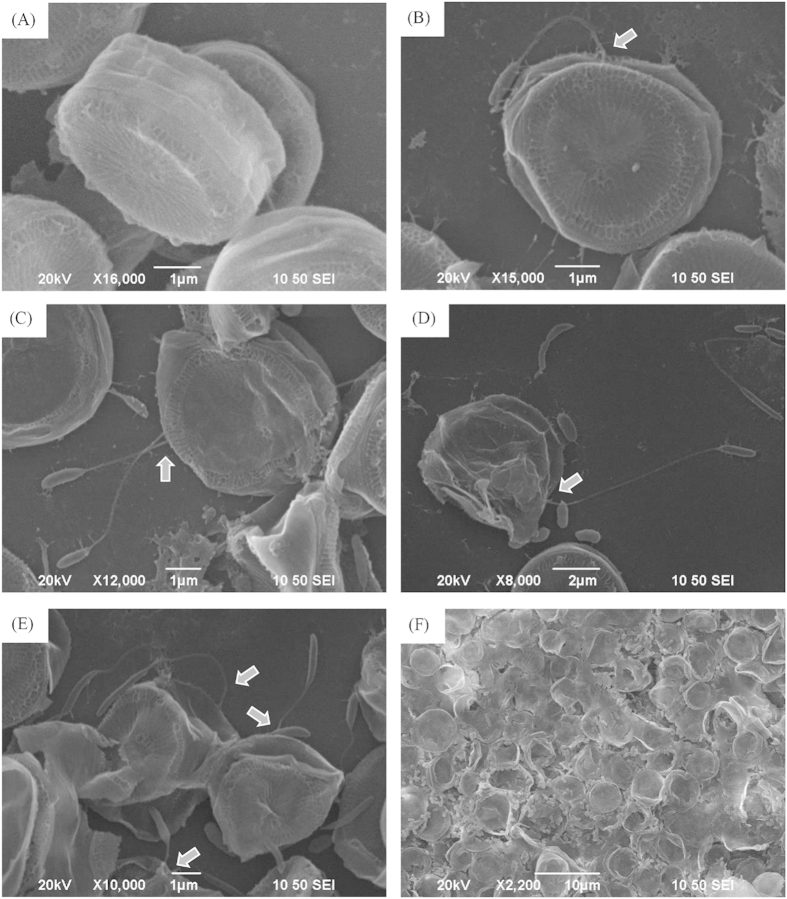
Scanning electron microscopy of interaction between strain LY03 and *Thalassiosira pseudonana* cells for 6 (**B**), 12 (**C**), 24 (**D**), 48 (**E**) and 72 h (**F**). (**A**) Control cells. Arrows show where the bacterial cells fastened onto the algal cells.

The interaction between strain LY03 and *T*. *pseudonana* cells was investigated as in Fig. 5. The algal cells in the control (Fig. 5A) showed normal cellular morphology, as well as clearly visible siliceous structures and patterns with thick-shells. After adding bacterial cells of strain LY03 into the algal culture, a very interesting interaction between bacteria and algae has been observed (Fig. 5B), in that strain LY03 could fasten itself onto the algal cells with its flagellum, sometimes several bacterial cells fastening on the same algal cells (Fig. 5C). At the same time, the algal cells which were adhered by bacterial cells were lysed and lost the siliceous shells and cellular structure, and eventually died (Fig. 5D–F). As shown in Fig. 5F, almost all the algal cells under the interaction of strain LY03 lysed and the broken siliceous shells showed that the algal cells were seriously damaged, so that strain LY03 posed a great threat to diatom growth.

**Figure 6:**
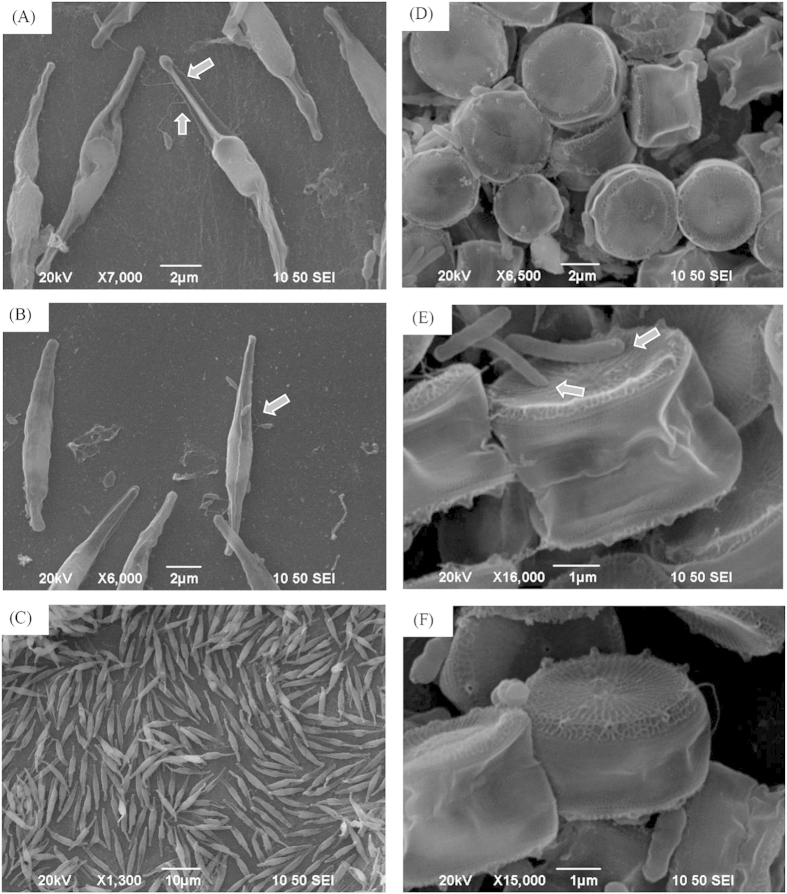
Scanning electron microscopy of interaction between strain LY03 and *P*. *tricornutum* cells for 24 (**A**), 48 (**B**) and 72 h (**C**) and the interaction characteristics between strain KACC 11467 and *T*. *pseudonana* cells for 24 (**D**), 48 (**E**) and 72 (**F**). Arrows show where the bacterial cells contacted with the algal cells.

The interaction between strain LY03 and *P*. *tricornutum* cells was also observed, as shown in Fig. 6A–C. We found that strain LY03 could again fasten its flagellum onto the body of the algal cells, using a method similar to the *T*. *pseudonana* cells, but the *P*. *tricornutum* cells always kept their normal cellular morphology, and were not affected by strain LY03. In terms of the interaction characteristic between strain KACC 11467 and *T*. *pseudonana* cells, the results in Fig. 6D–F showed that bacterial cells of strain KACC 11467 attached on the surface of *T*. *pseudonana* cells, but they were not influenced by them during the whole treatment procedure, and maintained normal growth state.

### Lysis of *T*. *pseudonana* under the effect of strain LY03

**Figure 7:**
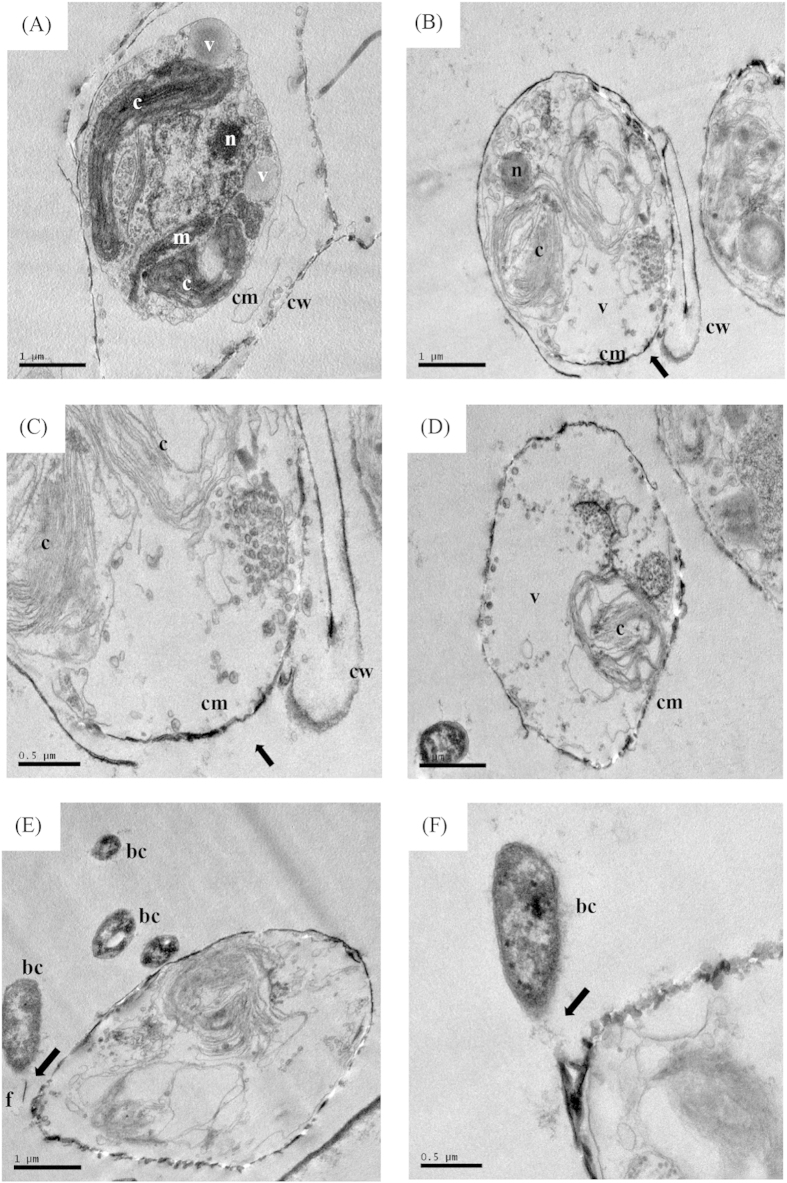
Transmission electron micrographs of the death processes in *T*. *pseudonana* treated with strain LY03 for 6 (**B,C**) and 24 h (**D–F**). (**A**) Control cells. (c: chloroplast; m: mitochondria; n: nucleus; cm: cell membrane; cw: cell wall; v- vacuole; bc: bacterial cells; f: flagellum). Bars (**A,B**,**D,E**) 1 μm; (**C**,**F**) 0.5 μm. Arrows show where the bacterial cells fastened onto the algal cells.

The TEM analysis revealed alterations in the ultrastructure of *T*. *pseudonana* under the algicidal effects of strain LY03, as well as an interaction between bacterial cells and algae (Fig. 7). As shown in Fig. 7A, the algal cells in the control showed normal cellular morphology with compact cell structure; dense cytoplasm protected by the cell membrane and the thick siliceous shell; and intact organelles including chloroplasts, mitochondria, nuclei arranged in order and tightly. Under the algicidal effect of strain LY03, the morphology and structure were seriously damaged, the siliceous frustules which contained chitin was degraded by chitinase, and obvious breaches could be found in the cell wall (Fig. 7B,C), as well as the destruction of chloroplasts and mitochondria. When the treatment time was prolonged, the cell wall was totally lysed, and large numbers of vacuoles occurred as well as degradation of the organelles (Fig. 7D). The TEM results also revealed the interaction between bacteria and algae, showing that the bacterial cells fastened onto the algal cells through their flagella and, at the same time, the algal cells lysed and died (Fig. 7E,F).

### Algicidal activity of different fractions in strain LY03

**Figure 8:**
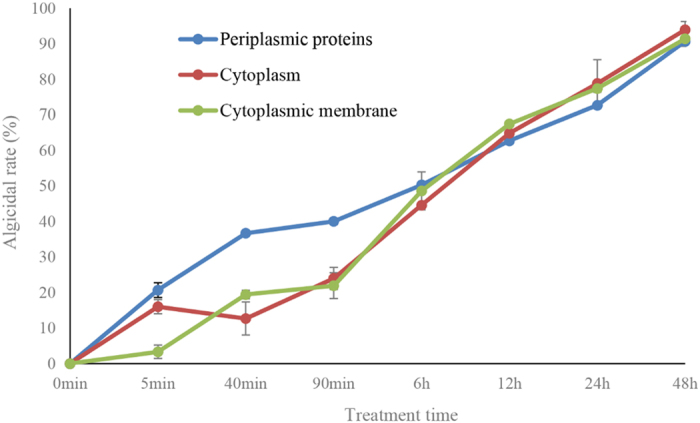
Algicidal activity of different fractions in bacterial cells of strain LY03. All error bars indicate the SE of the three biological replicates.

Periplasmic proteins, cytoplasm and cytoplasmic membrane were extracted from bacterial cells of strain LY03, and the algicidal activity of these different fractions on *T*. *pseudonana* cells were determined in Fig. 8. Within 5 min of treatment time, there showed algicidal activity in periplasmic proteins, cytoplasm, the algicidal rates reached to 20.7 and 16.0%, respectively. The algicidal activity in periplasmic proteins continued to rise, algicidal rate reached 50.3% within 6 h treatment, and 90.7% within 48 h treatment. The algicidal rate in the cytoplasm decreased within 40 min treatment. However, it has increased again during 90 min to 48 h of treatment time, the algicidal rate reached 94.0% within 48 h treatment. We also found algicidal activity in the cytoplasmic membrane within 40 min of treatment time, when the algicidal rate reached 19.4%, and it continued to increase to 91.4% within 48 h of treatment.

### Characteristic of intracellular algicidal substances

**Figure 9:**
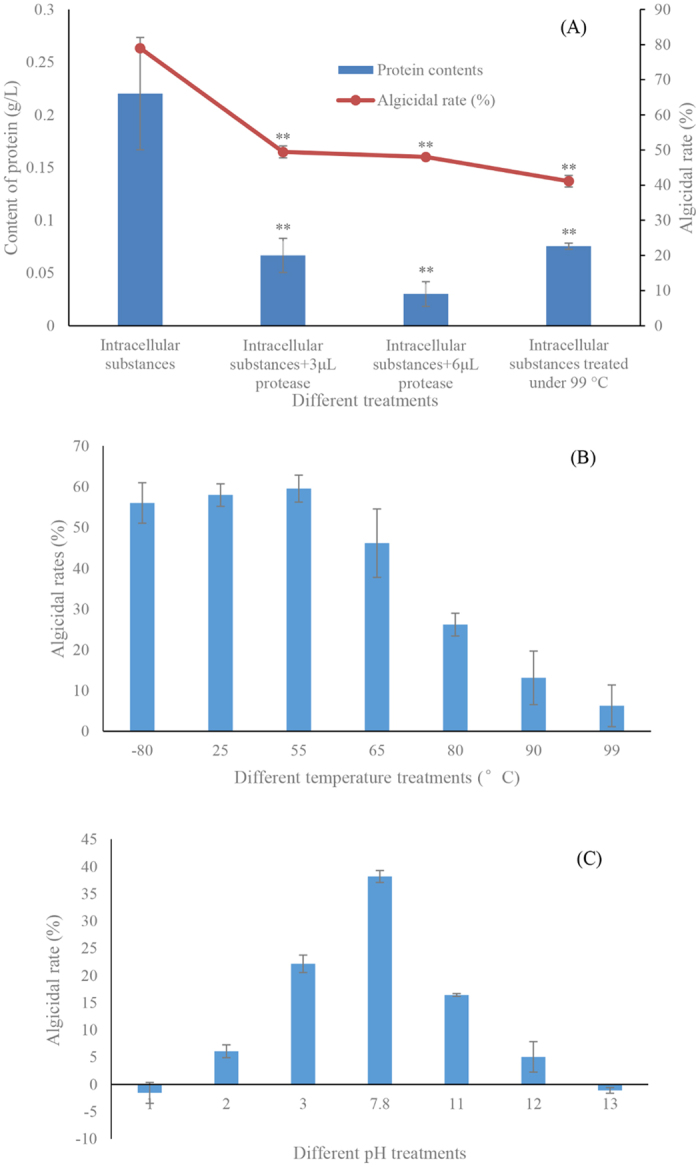
Algicidal activity of intracellular substances after treatment with protease (**A**) different temperature (**B**) and different pH (**C**). All error bars indicate the SE of the three biological replicates. * represents a statistically significant difference of *p* < 0.05 when compared to the control; ** represents a statistically significant difference of *p* < 0.01.

To identify the characteristics of intracellular algicidal substances, protease treatments, different temperature treatments and different pH treatments were used to determine the algicidal activity (Fig. 9). After treatment with protease (3 and 6 μl), the contents of intracellular protein obviously (*p* < 0.01) decreased when compared to the control (Fig. 9A). In addition to this decline of algicidal activity compared to control, the algicidal rate in the protease treatment groups decreased to 49.5 and 48.0%, while the algicidal rate in the control reached 79.0%. The intracellular protein content and algicidal rate after being heated at 99 °C also significantly (*p* < 0.01) decreased when compared to the control. The algicidal activity of intracellular substances after being treated under different temperatures showed clear distinctions (Fig. 9B), the algicial activity remained stable in the −80 to 55 °C treatment groups, almost reaching 60%. However, the algicidal rate began to decrease after heating at 65 °C or higher, decreasing to 46.2% in the 65 °C treatment group. The algicidal rate continued decreasing along with the increase of temperature, and decreased to only 6.3% in the 99 °C treatment group. As shown in Fig. 9C, the algicidal activity of intracellular substances after treatment with different pH values was investigated, an obvious decrease in low or high pH, with the algicidal rate decreasing to 22.1 and 16.4% when the pH value was 3 and 11. Especially pH value was 1 or 13, there showed no algicidal activity on algal cells, prompting algal growth instead.

## Discussion

Chitinase producing bacteria play an important function in the aquatic environment based on their extensive involvement in energy cycling and nutrient flow^15^, there have been a large number of studies to confirm that microorganisms, particularly bacteria, constitute one of the major sources of chitinase, which caused great interest because of broad applications in antibacterial and antifungal treatments as a source of medicines, in health foods and animal feeds^16^,^17^,^18^. However, the effect of chitinase producing bacteria on marine phytoplankton containing chitin is unclear, thus the interaction between chitinase producing bacteria and algae has important research value in the protection and utilization of algal resources.

The ability to produce chitinase in *C. prasina* LY03 and *C. koreensis* KACC 11467 is confirmed^10^,^19^, and these were chosen as the experimental bacterial strains in this study. All diatoms except *P*. *tricornutum* contain chitin in their siliceous shells^20^, and *P*. *tricornutum* and *T*. *pseudonana* (both with clear genome information) are almost always chosen as model diatoms^21^,^22^. Therefore, to study the interaction between chitinase producing bacteria and diatoms, we selected the two model organisms, *P*. *tricornutum* and *T*. *pseudonana* for this investigation.

To determine the algicidal effect of chitinase producing bacteriument marine algae with or without chitin, we investigated the algicidal range of strain LY03. The results showed that strain LY03 exhibited algicidal activity on all the diatoms with chitin, as well as the dinoflagellates. Strain LY03 had no effect on *P*. *tricornutum* or other algae which did not contain chitin in the exoskeleton. Brunner *et al.* have reported that the cell walls of *T.pseudonana* contain an internal, organic network consisting of crosslinked chitin fibers^23^. Ehrlich *et al.* and Durkin *et al.* have demonstrated the presence of chitin in diatoms and discussed its potential as scaffolding as well as a template material in siliceous cell walls of diatoms^24^,^25^. Ruiz-Herrera *et al.* also reported that chitin seems to be restricted to eukaryotes, and is present in members of all the “crown kingdoms”, which include Fungi, Plantae, Animalia, Alveolates (dinoflagellates), and Stramenopiles (diatoms)^26^. Therefore, since strain LY03 only showed algicidal activity on chitin containing algae, such as diatoms or dinoflagellates, the algicidal activity should have a certain relationship with chitinase which is produced by strain LY03. We also studied the algicidal effect of strain KACC 11467 which also has the ability to produce chitinase and which belongs to the same genus as strain LY03. The results turned out to be different from expected ones, and since strain KACC 11467 showed no algicidal activity on *T*. *pseudonana*, we speculated that there were other algicidal factors. To further explore the algicidal mechanism, we measured the algicidal mode of strain LY03. Both the bacterial culture and bacterial cells of strain LY03 showed high algicidal activity on algal cells. However, the bacterial supernatant did not have any algicidal effect. Therefore, the algicidal ability of strain LY03 was mainly due to the bacterial cells themselves, and the algicidal mode of strain LY03 was a direct-way. Kang *et al.* isolated one bacterium HYK0203-SK02, which showed strong algicidal effect on the growth of *Stephanodiscus hantzschii*, and determined that the algicidal substance was localized in the cytoplasmic membrane^27^. The bacterial cells of strain LY03 exhibited high algicidal activity on the growth of diatoms, but bacterial cells had no effect on *P*. *tricornutum* and other algae which did not contain chitin in the cells, and so chitinase might also play an important role in inhibiting algal growth.

To make sure whether the algicidal activity of strain LY03 was from bacterial cells or intracellular substances, we first investigated the bacterial viability after bacterial cells were added into f/2 medium for different times. Bacterial viability was measured using FCM after the bacterial cells were stained with PI. The fluorescence dye PI cannot pass through complete cell membrane of normal cells, whereas the cell membrane of dying or dead cells lose their integrity and the change of cell membrane permeability allows the cells to be stained by PI^28^. Therefore, we could use FCM to analyze the cells with PI staining, which implied bacterial viability. In the control, almost all the bacterial cells had strong vitality, and only 0.12% of the bacterial cells were stained with PI. We measured the ratio of bacterial cells stained with PI after the cells were cultured in f/2 for 24 h, and 4.39% of the cells were stained by PI, which indicated that only tiny amounts of bacterial cells lost their viability, and most of the cells (95%) remained viable. We also analyzed the ratio of bacterial cells stained with PI after the bacterial cells were cultured in f/2 medium for 48 and 72 h, and the results indicated that only 3.17 and 4.31% of the bacterial cells were stained by PI. Therefore, we concluded that bacterial cells of strain LY03 could maintain bacterial viability in algal cultures, and were involved in lysing algal cells.

Since bacterial cells contributed to algicidal activity, to further confirm the algicidal effect of bacterial cells, the chemotactic ability of strain LY03 was investigated. Chemotactic ability is vital to the capacity for bacteria to degrade chitin. Li and Roseman reported that chitinase producing bacteria possessed chitin catabolic cascade including extracellular chitinases and chemotaxis systems specific for chitin oligosaccharides^4^. The chemotactic ability of strain LY03 and KACC 11467 were investigated in our study, and the results confirmed that strain LY03 showed chemotactic ability to *T*. *pseudonana*, as well as *P*. *tricornutum*. We deduced that since algal cells could secrete organic nutrients through photosynthesis, and that bacterial cells had a chemotactic potential towards nutrients in order to achieve the purpose of self-growth, therefore, strain LY03 could be chemotactic to any materials which were beneficial to itself. The chemotactic ring of strain KACC 11467 also proved strain KACC 11467 had chemotactic ability. Wadhams and Armitage reported bacteria could respond to changing environments via bacterial chemotaxis, which relied on flagellar switching^29^. The chemotactic ability of chitinase producing bacteria could be conducive to realizing the degradation of chitin.

Understanding the interaction between bacteria and algae is helpful in the study of the algicidal mechanism, and so we observed the interaction process through SEM analysis. We have observed a novel interaction between strain LY03 and diatoms. The flagella of strain LY03 were fixed tightly on the algal cell but, at the same time, the bacterial cells made no contact with the algal cells. Although there was always constant fluctuation of the water, the bacterial cells of strain LY03 connected closely with the algae through the linking of flagella. We also studied the interaction between strain KACC 11467 and *T*. *pseudonana*, and found that bacterial cells of strain KACC 11467 could contact with the surface of the algal cells by means of its chemotactic ability. However, the connection was not close and tight, since strain KACC 11467 could separate itself from the algal cells at any time. Comparing the interaction characteristics of strain LY03 and KACC 11467, we concluded that the algicidal ability of strain LY03 provided a great relationship with a special interaction mode, and that the tight connection with the algal cells suggested the implementation of the algicidal effect. The interaction between strain LY03 and *P*. *tricornutum* was also investigated, and surprisingly the interaction between strain LY03 and *P*. *tricornutum* was similar to the interaction between strain LY03 and *T*. *pseudonana*, in that strain LY03 could fasten itself on *P*. *tricornutum* through flagella, but showed no algicidal effect on *P*. *tricornutum*. Therefore, there must be other reasons related to the algicidal effect of strain LY03.

To investigate other algicidal reasons, we extracted different fractions (periplasmic proteins, cytoplasm and cytoplasmic membrane) from the bacterial cells of strain LY03. As shown in the results, all the fractions showed algicidal activity on algal cells, and the algicidal effect was very rapid. Periplasmic proteins and cytoplasm expressed algicidal ability within only 5 min, and the algicidal rate reached 90% with 48 h of treatment. There could be algicidal substance secreted inside the bacterial cells, which could be released to the extracellular environment and showed algicidal effect on the algal cells at a close range with the help of flagellar fixation. We studied the algicidal effect on *T*. *pseudonana* cells through TEM analysis, found that the algal cell wall was degraded by strain LY03, and the morphology and structure of the chloroplasts and mitochondria were seriously damaged leading to cell lysis and death. The flagella of bacterial cells were found to be inside the algal cells as well as causing their lysis.

The algicidal effect of strain LY03 on diatoms was based on a combination of bacterial flagellar fixation and intracellular algicidal substances. To further understand the characteristics of the intracellular substances, we investigated the algicidal effect of intracellular substances after protease treatment, at different temperatures and pH values. The algicidal effect of intracellular substances after treatment with protease has apparently decreased. Likewise decreased the protein contents implying that the intracellular algicidal substances could be protein substances, and could be decomposed by protease. The different ranges of temperature treatment showed that intracellular algicidal substances decreased after the temperature increased to 65 °C, and lost activity after further temperature increase. The algicidal activity reached its highest level when the temperature was 55 °C, and there are many reports that chitinase activity would reached to highest level at 55 °C^30^,^31^. Different pH treatment groups showed that intracellular algicidal substances lost their algicidal activity either at high or low pH, so that the intracellular substances were sensitive to pH and exhibited pH instability. In general, intracellular substances should be protein substances, combined with the algicidal range which showed an algicidal effect only on chitin containing algae, as well as other characteristics, suggested that the only plausible explanation is that the intracellular algicidal substances seemed to be chitinase.

The interaction between chitinase producing bacteria and diatoms was determined for the first time in this study, and the algicidal mode, range, chemotactic ability, interaction characteristics, intracellular algicidal substance characteristics were also investigated. The algicidal mechanism was that bacterial cells of LY03 could show chemotaxis to diatom cells, and fasten tightly to algal cells with their flagella, and then produce chitinase to degrade the algal cell walls, and eventually kill the algal cell. This is the first report of the functional mechanism of direct algicidal bacteria with fast and efficient algicidal effect on diatoms. Further, studies should focus on the determination of the type of protease produced by strain LY03.

## Additional Information

**How to cite this article**: Li, Y. *et al.* Chitinase producing bacteria with direct algicidal activity on marine diatoms. *Sci. Rep.* **6**, 21984; doi: 10.1038/srep21984 (2016).

## Supplementary Material

Supplementary Information

## References

[b1] Kumar M. N. V. R. A review of chitin and chitosan applications. React. Funct. Polym. 46, 1–27 (2000).

[b2] Tharanathan R. N. & Kittur F. S. Chitin—the undisputed biomolecule of great potential. Crit. Rev. Food Sci. 43, 61–87 (2003). 10.1080/10408690390826455 12587986

[b3] Keyhani N. O. & Roseman S. Physiological aspects of chitin catabolism in marine bacteria. BBA-Gen. Subjects 1473, 108–122 (1999). 10.1016/s0304-4165(99)00172-5 10580132

[b4] Li X. & Roseman S. The chitinolytic cascade in *Vibrios* is regulated by chitin oligosaccharides and a two-component chitin catabolic sensor/kinase. P. Natl. Acad. Sci. USA 101, 627–631 (2004). 10.1073/pnas.0307645100 PMC327198 14699052

[b5] Barton A. D., Dutkiewicz S., Flierl G., Bragg J. & Follows M. J. Patterns of diversity in marine phytoplankton. Science 327, 1509–1511 (2010). 20185684 10.1126/science.1184961

[b6] Amin S. A., Parker M. S. & Armbrust E. V. Interactions between Diatoms and Bacteria. Microbiol. Mol. Biol. R. 76, 667–684 (2012). 10.1128/MMBR.00007-12 PMC3429620 22933565

[b7] Thamatrakoln K. *et al.* Death-specific protein in a marine diatom regulates photosynthetic responses to iron and light availability. P. Natl. Acad. Sci. USA 110, 20123–20128 (2013). 10.1073/pnas.1304727110 PMC3864291 24277817

[b8] Rings A., Lücke A., Schleser G. H., Rings A. & Lücke A. A new method for the quantitative separation of diatom frustules from lake sediments. Limnol. Oceanogr. Meth. 2, 25–34 (2004).

[b9] Bidle K. D. & Azam F. Accelerated dissolution of diatom silica by marine bacterial assemblages. Nature 397, 508–512 (1999).

[b10] Li Y. *et al.* *Chitinimonas prasina* sp. nov., isolated from lake water. Int. J. Syst. Evol. Micr. 64, 3005–3009 (2014). 10.1099/ijs.0.061234-0 24907265

[b11] Guillard R. R. In Culture of marine invertebrate animals 29–60 (Springer, 1975).

[b12] Zhang B. *et al.* *Streptomyces alboflavus* RPS and its novel and high algicidal activity against harmful algal bloom species *Phaeocystis globosa*. PloS one 9, e92907 (2014). 24675867 10.1371/journal.pone.0092907 PMC3968035

[b13] Tanaka Y., Yamaguchi N. & Nasu M. Viability of *Escherichia coli* O157: H7 in natural river water determined by the use of flow cytometry. J. Appl. Microbiol. 88, 228–236 (2000). 10735990 10.1046/j.1365-2672.2000.00960.x

[b14] Jung S., Kim B. H., Katano T., Kong D. S. & Han M. S. *Pseudomonas fluorescens* HYK0210‐SK09 offers species‐specific biological control of winter algal blooms caused by freshwater diatom *Stephanodiscus hantzschii*. J. Appl. Microbiol. 105, 186–195 (2008). 18266701 10.1111/j.1365-2672.2008.03733.x

[b15] Svitil A. L., Chadhain S., Moore J. A. & Kirchman D. L. Chitin degradation proteins produced by the marine bacterium *Vibrio harveyi* growing on different forms of chitin. Appl. Environ. Microb. 63, 408–413 (1997). 10.1128/aem.63.2.408-413.1997 PMC1389511 16535505

[b16] Bhattacharya D., Nagpure A. & Gupta R. K. Bacterial chitinases: properties and potential. Crit. Rev. Biotechnol. 27, 21–28 (2007). 17364687 10.1080/07388550601168223

[b17] Cottrell M. T., Moore J. A. & Kirchman D. L. Chitinases from uncultured marine microorganisms. Appl. Environ. Microb. 65, 2553–2557 (1999). 10.1128/aem.65.6.2553-2557.1999 PMC91377 10347042

[b18] Singh P. P., Shin Y. C., Park C. S. & Chung Y. R. Biological control of Fusarium wilt of cucumber by chitinolytic bacteria. Phytopathology 89, 92–99 (1999). 18944809 10.1094/PHYTO.1999.89.1.92

[b19] Kim B.-Y. *et al.* *Chitinimonas koreensis* sp. nov., isolated from greenhouse soil in Korea. Int. J. Syst. Evol. Micr. 56, 1761–1764 (2006). 10.1099/ijs.0.64163-0 16902004

[b20] Montsant A., Jabbari K., Maheswari U. & Bowler C. Comparative genomics of the pennate diatom *Phaeodactylum tricornutum*. Plant Physiol. 137, 500–513 (2005). 15665249 10.1104/pp.104.052829 PMC1065351

[b21] Bowler C. *et al.* The *Phaeodactylum* genome reveals the evolutionary history of diatom genomes. Nature 456, 239–244 (2008). 18923393 10.1038/nature07410

[b22] Armbrust E. V. *et al.* The genome of the diatom *Thalassiosira pseudonana*: ecology, evolution, and metabolism. Science 306, 79–86 (2004). 15459382 10.1126/science.1101156

[b23] Brunner E. *et al.* Chitin-based organic networks: an integral part of cell wall biosilica in the diatom *Thalassiosira pseudonana*. Angew. Chem. Int. Ed. 121, 9724–9727 (2009). 10.1002/anie.200905028 19924754

[b24] Ehrlich H. & Witkowski A. In Evolution of Lightweight 39–58 (Springer, 2015).

[b25] Durkin C. A., Mock T. & Armbrust E. V. Chitin in diatoms and its association with the cell wall. Eukaryot. cell 8, 1038–1050 (2009). 19429777 10.1128/EC.00079-09 PMC2708456

[b26] Ruiz‐Herrera J., Manuel González‐Prieto J. & Ruiz‐Medrano R. Evolution and phylogenetic relationships of chitin synthases from yeasts and fungi. FEMS Yeast Res. 1, 247–256 (2002). 12702327 10.1111/j.1567-1364.2002.tb00042.x

[b27] Kang Y. H., Kim J. D., Kim B. H., Kong D. S. & Han M. S. Isolation and characterization of a bio‐agent antagonistic to diatom, Stephanodiscus hantzschii. J. Appl. Microbiol. 98, 1030–1038 (2005). 15836471 10.1111/j.1365-2672.2005.02533.x

[b28] Li Y. *et al.* Toxicity of algicidal extracts from *Mangrovimonas yunxiaonensis* strain LY01 on a HAB causing *Alexandrium tamarense*. J. Hazard. Mater. 278, 372–381 (2014). 24997253 10.1016/j.jhazmat.2014.06.032

[b29] Wadhams G. H. & Armitage J. P. Making sense of it all: bacterial chemotaxis. Nat. Rev. Mol. Cell Bio. 5, 1024–1037 (2004). 15573139 10.1038/nrm1524

[b30] Chen J. P. & Chang K. C. Immobilization of chitinase on a reversibly soluble–insoluble polymer for chitin hydrolysis. J. Chem. Technol. Biot. 60, 133–140 (1994). 10.1002/jctb.280600204 7764962

[b31] Bhushan B. & Hoondal G. S. Isolation, purification and properties of a thermostable chitinase from an alkalophilic *Bacillus* sp. BG-11. Biotechnol. Lett. 20, 157–159 (1998).

