# Supplementary material for: Chitinase producing bacteria with direct algicidal activity on marine diatoms
Source: Sci Rep. 2016 Feb 23;6:21984. doi: 10.1038/srep21984 (PMC4763246; doi:10.1038/srep21984)
Supplement: Supplementary Information [file srep21984-s1.doc]

**Supplement data**

**Chitinase producing bacteria with direct algicidal activity on marine diatoms**

Yi Li^1, 2※^, Xueqian Lei^1※^, Hong Zhu^1^, Huajun Zhang^1^, Chengwei Guan^1, 4^, Zhangran Chen^1^, Wei Zheng^1^, Lijun Fu^3^*, Tianling Zheng^1^*

*1, State Key Laboratory of Marine Environmental Science and Key Laboratory of MOE for Coast and Wetland Ecosystems, School of Life Sciences, Xiamen University, Xiamen 361005, China*

*2, College of Life Sciences, Henan Normal University, Xinxiang, 453007, China*

*3, Department of Environment and Life Science, Putian University, Putian 351100, China*

*4,Tobacco Science Institute of Jiangxi Province, Nanchang 330000, China*

^※^These authors contributed equally to this work and should be regarded as co-first authors.

* Corresponding author: Tianling Zheng. E-mail: [wshwzh@xmu.edu.cn](mailto:wshwzh@xmu.edu.cn); LijunFu. E-mail:lijun-fu@sina.com


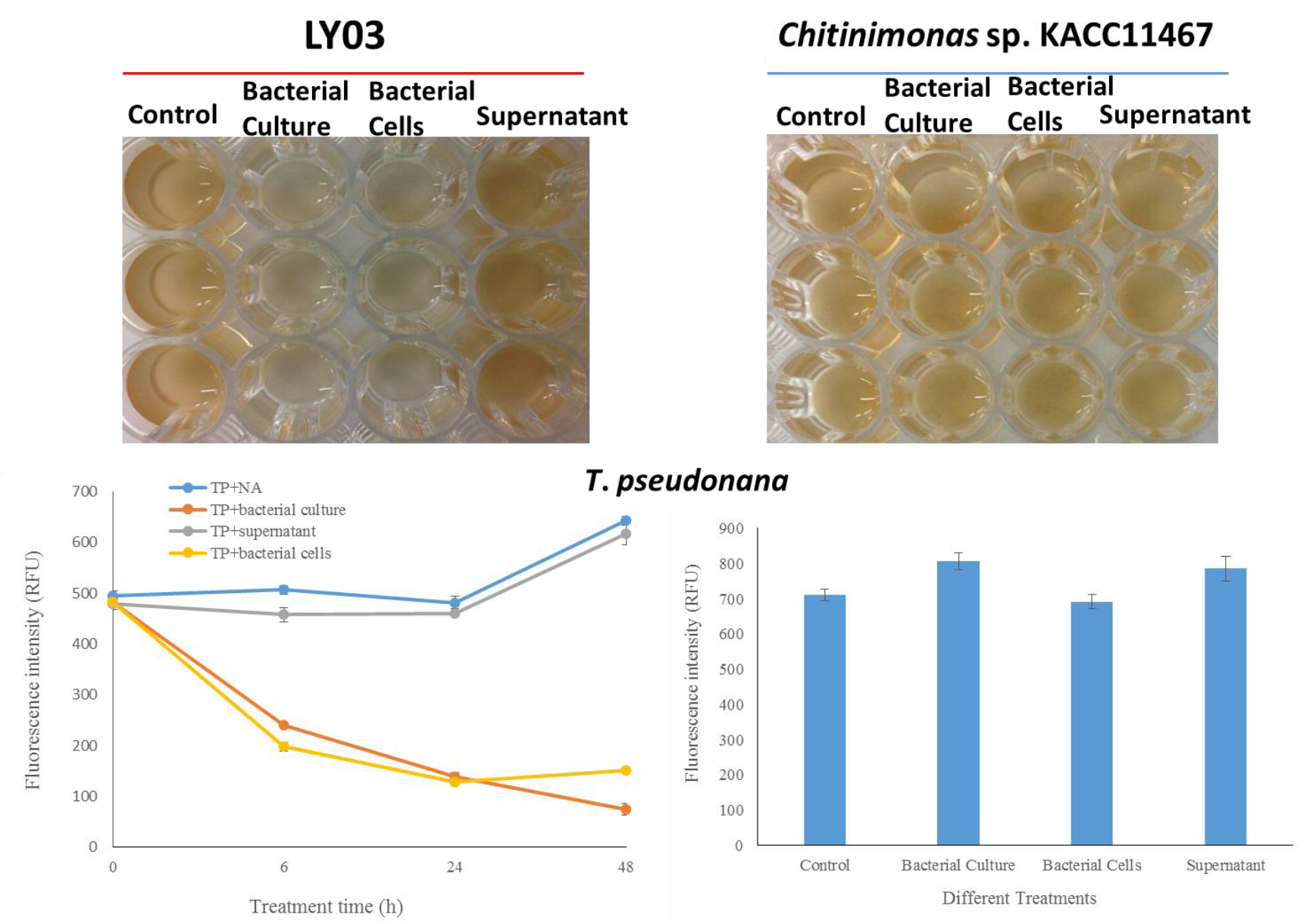


Figure S1 Algicidal activity of *C*. *prasina* LY03 and *C. koreensis* KACC 11467 on *T. pseudonana*
